# Identification and Replication of Three Novel Myopia Common Susceptibility Gene Loci on Chromosome 3q26 using Linkage and Linkage Disequilibrium Mapping

**DOI:** 10.1371/journal.pgen.1000220

**Published:** 2008-10-10

**Authors:** Toby Andrew, Nikolas Maniatis, Francis Carbonaro, S. H. Melissa Liew, Winston Lau, Tim D. Spector, Christopher J. Hammond

**Affiliations:** 1 Twin Research and Genetic Epidemiology, King's College London, St Thomas' Hospital, London, United Kingdom; 2 Department of Genetics, Evolution and Environment, University College London, London, United Kingdom; 3 West Kent Eye Centre, Bromley Hospitals NHS Trust, Orpington, London, United Kingdom; The University of Queensland, Australia

## Abstract

Refractive error is a highly heritable quantitative trait responsible for considerable morbidity. Following an initial genome-wide linkage study using microsatellite markers, we confirmed evidence for linkage to chromosome 3q26 and then conducted fine-scale association mapping using high-resolution linkage disequilibrium unit (LDU) maps. We used a preliminary discovery marker set across the 30-Mb region with an average SNP density of 1 SNP/15 kb (Map 1). Map 1 was divided into 51 LDU windows and additional SNPs were genotyped for six regions (Map 2) that showed preliminary evidence of multi-marker association using composite likelihood. A total of 575 cases and controls selected from the tails of the trait distribution were genotyped for the discovery sample. Malecot model estimates indicate three loci with putative common functional variants centred on *MFN1* (180,566 kb; 95% confidence interval 180,505–180, 655 kb), approximately 156 kb upstream from alternate-splicing *SOX2OT* (182,595 kb; 95% CI 182,533–182,688 kb) and *PSARL* (184,386 kb; 95% CI 184,356–184,411 kb), with the loci showing modest to strong evidence of association for the Map 2 discovery samples (*p*<10^−7^, *p*<10^−10^, and *p* = 0.01, respectively). Using an unselected independent sample of 1,430 individuals, results replicated for the *MFN1* (*p* = 0.006), *SOX2OT* (*p* = 0.0002), and *PSARL* (*p* = 0.0005) gene regions. *MFN1* and *PSARL* both interact with *OPA1* to regulate mitochondrial fusion and the inhibition of mitochondrial-led apoptosis, respectively. That two mitochondrial regulatory processes in the retina are implicated in the aetiology of myopia is surprising and is likely to provide novel insight into the molecular genetic basis of common myopia.

## Author Summary

Successful gene mapping strategies for common disease continue to require careful consideration of basic study design with the advent of genome-wide association studies. Here, we take advantage of prior information that the heritability of the quantitative trait myopia in the general population is high and shows evidence of replicated linkage to chromosome 3q26. Based on this, we conducted a fine map linkage disequilibrium association study for the region, using a high-resolution genetic map derived from population-based HapMap Phase II data. For analysis, we used efficient multi-locus tests of association using single nucleotide polymorphism markers genotyped for our sample data and placed on the genetic map measured in linkage disequilibrium units. We followed up preliminary evidence of association for the discovery samples with further genotyping in the same samples to improve the model location estimates for the common functional variants we identified. Three locations were replicated using an independent sample. Two of the identified genes are likely to play an unexpected role in myopia with both pivotal in the healthy housekeeping metabolism of retinal mitochondria. Both proteins interact with OPA1, with nonsynonymous *OPA1* mutations causing the unrelated Mendelian disease Autosomal Dominant Optic Atrophy (ADOA) by triggering mitochondrial-led retinal ganglia cell apoptosis.

## Introduction

Myopia is the most common eye disorder, affecting an estimated 36% of adults over 20 years in the United States [1] and up to 61% in East Asia [2]. Myopia is a significant cause of vision loss [3], and is becoming the most common single cause of blindness in the working age population [4]. Refractive error, measured in spherical equivalent (SE) diopters, is a quantitative trait influenced by multiple genetic and environmental factors. Myopia develops as a result of structural changes in the eye, particularly ocular axial length elongation, causing parallel rays of light to be focused in front of the retina, forming a blurred image. There are animal models for myopia development [5], but the mechanisms responsible for detecting lack of focus and the signalling pathways from the retina to the choroid and ultimately to the sclera to induce eye growth, are not well-understood.

Epidemiological studies have identified close visual work (and correlates of this such as hours spent reading, education and IQ) to be a significant risk factor for myopia development in children and that outdoor activity appears to be protective [2]. Twin studies also consistently demonstrate a large heritability for individual variation in refractive error around a specified population mean, ranging from 75–94% [6]. We previously described a genome-wide linkage analysis using autorefractor data for 221 dizygotic (DZ) female twin pairs, which identified 4 possible susceptibility loci, MYP7 on chromosome 11p13, MYP8 on chromosome 3q26, MYP9 on chromosome 4q12, and MYP10 on chromosome 8p23 [7]. However, to date, these loci have not been replicated, and no known myopia susceptibility genes have been identified [8].

The aims of this study were twofold. The first was to replicate the linkage signals at these four loci using an independent sample of DZ twins to the original study, using measures of refractive error (optician prescription) obtained via a postal questionnaire. The second aim was to conduct a follow-up association study of the genomic region with strongest evidence of replicated linkage, using linkage disequilibrium mapping to identify possible susceptibility genes and to replicate results using an independent sample.

## Results

### Subjects

Refraction data, either from autorefractor or postal prescription, were available for 4273 UK twin subjects (1716 complete pairs) with SE data. Overall, the SE mean (between sib-pair standard deviation) was −0.29D (2.36), range −20D to +8.75D with an inter-quartile range of −1.06D to +1.125D and 26% of the subjects were myopic using a threshold of SE< = −1D. A total 1846 autorefractor measures (915 complete pairs) and 997 postal prescriptions (485 pairs) were available for the discovery phase of this study and an independent sample of 1430 twins for replication (Table 1). The mean age of subjects was 53.7 years (SD 13.1), range 16–82 years, and 90.3% of subjects were female. The high proportion of women is the result of long-term recruitment of female volunteers for study of phenotypes such as osteoporosis.

**Table 1:** Phenotype and genotype data for discovery (Map 1 and Map 2) and replication samples.

Spherical equivalence	Mean	SD	Range		n		
			Min	Max	Total	Pairs	
All twins	−0.29	2.36	−20.0	8.75	4273	1716	tick
Linkage (replication)	−0.55	2.34	−20.0	8.1	997	485	tick
Association (discovery Maps 1 & 2)	−0.08	2.28	−17.8	7.3	1846	915	tick
*Cases*	−*3.92*	*2.43*	−*17.8*	−*1*	*293*	-	tick
*Controls*	*2.88*	*1.46*	*1.0*	*7.8*	*282*	-	tick
*Total*					*575*		
Association (replication)	−0.23	2.24	−12.6	8.75	1430	316	tick
Age (All twins)	53.7	13.1	16.1	82.1	4273	1716	tick
Age (linkage replication)	54.1	9.7	23.2	77.2	997	485	
Age (assoc. discovery)	58.8	11.9	17.8	81.7	575	-	
Age (assoc. replication)	52.9	12.5	16.1	82.1	1430	316	

Genotype 3q26 data	# SNPs		# Samples				
	Total	QC	Case	Control	Total	Pairs	
Association (discovery)							
Map 1	1920	1800	205	216	421	-	tick
Map 2	382	307	225	218	443	-	tick
*Total*	*2302*	*2107*	*293*	*282*	*575*	-	
Association (replication)		374	-	-	1430	316	tick

Phenotype summary: Refraction data (autorefractor and postal) for all samples (All twins), linkage replication (postal samples) and association samples (discovery = Map 1 and Map 2; replication). Association discovery = total autorefractor data used to select discovery case-controls; case-control = total number of Map 1 and Map 2 samples; Association replication = independent twin sample with autorefractor/postal spherical equivalence and Hap300 data; Pairs = number of twin pairs with complete refraction data for both siblings; SD = standard deviation (for unrelated case-controls) and between sib-pair standard deviation (for related samples). Genotype summary: Numbers of SNPs and samples genotyped; Association discovery Total = Map 1 and Map 2 coverage (total SNPs and samples); QC = Quality control; Association replication = Illumina Hap300 SNPs.

### Linkage Mapping

**Figure 1:**
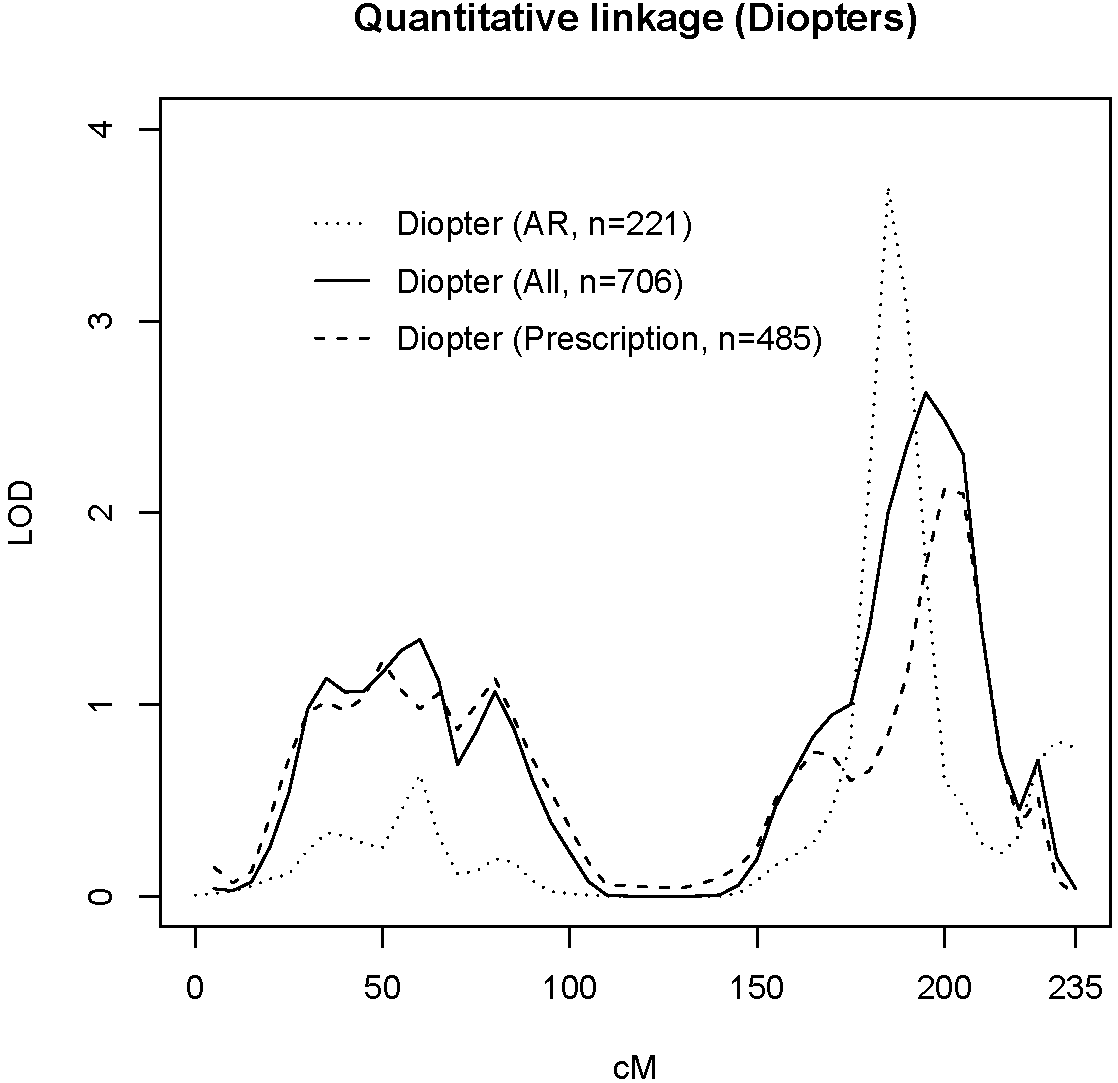
Evidence of replicated linkage to 3q26 region. AR (dotted line) = Autorefractor discovery sample for 221 DZ twin pairs (LOD 3.7) based upon previously published data [7]; Prescription (dashed line) = Replication sample using 485 DZ twin pairs with complete postal prescription data (LOD 2.12); All (solid line) = pooled linkage analysis for 706 DZ twin pairs (LOD 2.63).

For this study, we attempted to replicate linkage to four loci previously reported by our group [7]. Figure 1 illustrates linkage peaks to 3q26 for a discovery sample using autorefractive data with LOD 3.7 (DZ twin pairs = 221) based upon previously published data [7] and replication sample using postal prescription data with LOD 2.12 (DZ pairs = 485). Combined linkage using pooled data gave LOD 2.63 (DZ pairs = 706).

Marginal evidence for replicated linkage using the original Généthon map and microsatellite data for independent samples was also observed for MYP7 (11p13) and MYP9 (4q26), but not for MYP10 (8p23). These loci are currently subject to further investigation.

### Association Mapping: Selection of Myopic Cases and Hyperopic Controls for Discovery Sample

Autorefractor rather than postal prescription data were used for discovery stage association mapping, since autorefractor data are observed to be more precise with a smaller standard deviation (AR total sib-pair SD = 2.48; postal SD = 2.76; p = 0.0006). Subjects measured using an autorefractor were measured in the same standardized manner with no transcription errors that tend to be associated with postal prescription data.

For the initial fine mapping study of the 3q26 region (Map 1), a total of 243 cases and 257 controls selected from myopic and hyperopic concordant sib-pairs, respectively, were defined from the lower and upper quartiles of the SE quantitative trait using 915 twin pairs with complete autorefractor data (see Materials and Methods). Seventy-nine of these had depleted DNA (46 with none and 33 samples with poor DNA quality or case-wise missing > = 30%), leaving 205 cases (myopic individuals with a myopic sibling) and 216 controls (hyperopic individuals with a hyperopic sibling), a total of 421 case-controls for the preliminary Map 1 analysis (Table 1B).

The Map 2 data contained 154 new samples and approximately 70% of the samples from Map 1, yielding a total of 443 case-controls. Hence a total of 575 cases and controls were genotyped for either the Map 1 (n = 421) or Map 2 (n = 443) discovery samples (Table 1B), with 289 samples genotyped for both. It was intended to genotype the same samples for Maps 1 and 2, but due to low DNA stock for some of the original Map 1 samples sent to Ellipsis for genotyping; new case/control samples with sufficient DNA stock were used to replace depleted Map 1 samples.

### LDU Maps and SNP Selection

**Figure 2:**
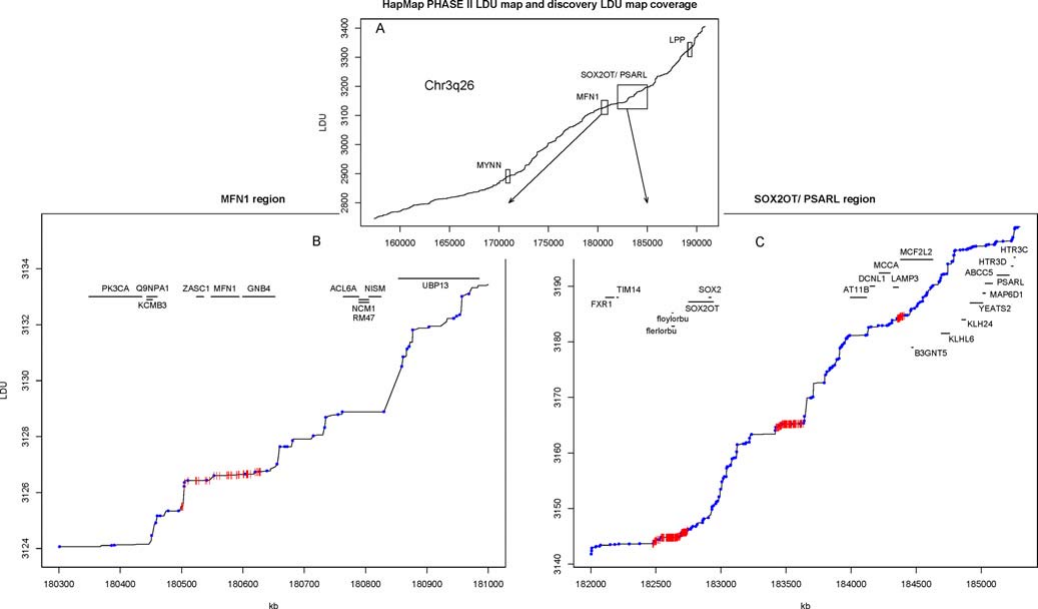
PHASE II LDU map and SNP sample marker coverage for the 3q26 region. A) PHASE II map linkage disequilibrium units (LDU) plotted against physical location (kb) with two replicated regions highlighted with a box and arrow. B) and C) illustrate the block-step LDU structure for the *MFN1* and *SOX2OT* gene regions and the increase in SNP selection density from Map 1 (blue points) to Map 2 (red lines).

Figure 2 illustrates the high-resolution linkage disequilibrium unit (LDU) map (Figure 2A), based upon 24,331 HapMap PHASE II SNPs for the 3q26 region (described in Materials and Methods), used to select informative markers for this study. The figure plots the relationship between cumulative genetic distance on the Y-axis (LDU) and physical location on the X-axis (kb). The LDU map provides detailed information on fine-scale linkage disequilibrium. The horizontal steps seen in Figures 2B and 2C represent regions of extended LD, while rapid increments in cumulative LDU represent regions of breakdown in LD, primarily due to recombination [9]. The LDU map for the entire 3q26 region used for this study is presented in Table S1.

For the Map 1 samples, we attempted to genotype a total of 2304 SNPs. After removing non-polymorphic SNPs (384 SNPs), SNPs with a call rate ≤90% (84), evidence of Hardy-Weinberg disequilibrium (38) and MAF< = 1% (0), a total of 1800 out of 1920 polymorphic SNPs remained for Map 1 analysis.

For the second stage of the association study (Map 2), in order to further refine the location of detected association, we genotyped an additional set of 382 SNPs for those LDU regions from Map 1 that showed evidence of association with myopic case-control status. Hence Map 2 had a high local LD resolution. After removing non-polymorphic Map 2 SNPs (33), SNPs with a call rate ≤90% (19), evidence of Hardy-Weinberg disequilibrium (20) and MAF< = 1% (7), a total of 307 SNPs remained for Map 2 analysis.

### Association Mapping

Map 1 provided preliminary multi-marker evidence of association to six gene regions (Figure 2), namely *MYNN* (p = 0.0028), *MFN1* (p = 0.02), upstream of *SOX2OT* (p = 0.0082), a “gene desert” region downstream from *SOX2OT* (between *SOX2* and *AT11B*; p = 0.009), *MCF2L2/PSARL* (p = 0.014) and *LPP* (p = 0.003), with each analytical LDU window spanning 939 kb, 672 kb, 965 kb, 753 kb, 944 kb and 465 kb, respectively. Additional Map 2 genotyping within Map 1 provided the same or increased evidence of association for the *MYNN* (p = 9.2×10^−5^), *MFN1* (p = 1.54×10^−8^), upstream of *SOX2OT* (p = 1.1×10^−11^), downstream of *SOX2OT* (p = 1.6×10^−5^) and *MCF2L2/PSARL* regions (p = 0.01), but not *LPP* (p = 0.03). Hence four of the regions provided statistically significant evidence of association at the discovery phase with a significance threshold of α = 10^−4^ (accounting for discovery multiple testing, see Materials and Methods), with the *MFN1* and upstream *SOX2OT* regions attaining genome-wide significance (α≈10^−8^).

For replication, we used an opportunistic sample in which we excluded all discovery twin samples (and their co-twins) from the TwinsUK register, to obtain 1430 individuals complete for autorefractor or postal SE and genotypes at 3q26 based upon the Illumina genome-wide Hap300 chip made available from other ongoing studies. Using quantitative tests of association, the same Malecot models and analytical LDU windows were fitted to the replication data. A significance threshold of α = 10^−2^ was used for the replication tests (see Materials and Methods).

All single-SNP allelic tests of association results (see Association Mapping, Materials and Methods) for Map 1, Map 2 and replication samples are presented in Tables S2, S3, and S4, respectively.

### MFN1 Region

**Figure 3:**
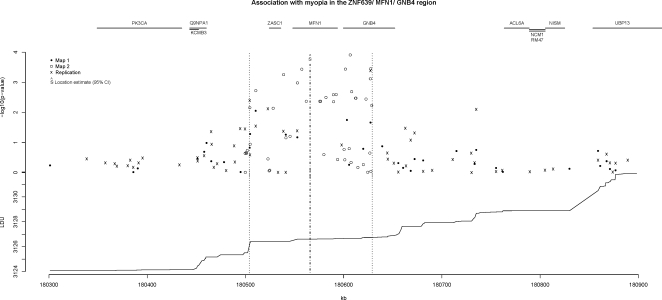
Association with myopia in the *ZNF639*, *MFN1*, *GNB4* region. Scatter plot of −log10(p-values) for individual markers vs. physical location in kb, with the top of the graph showing gene co-ordinates and the lower graph, cumulative LDU vs. physical location (both graphs on the same kb scale). The vertical dotted lines represent the location parameter estimate, Ŝ_kb_, and 95% confidence intervals. See Figure S1 for pair-wise LD plot of region.

Based on the Malecot model, the most likely physical location for a putative common functional variant in the *MFN1* region was estimated to be at 180,566 kb with the 95% confidence interval ranging from 180,505–180,655 kb (Map 2, Table 2). The variant location estimate at 180,566 kb lies in exon 7 of the *MFN1* gene (180,548–180,594 kb, approximately 45.5 kb in length), but the confidence interval for this estimated location also includes the genes *ZNF639* (ZASC1), *MFN1* and *GNB4* (Figure 3).

**Table 2:** Localization of genetic variants to the *ZNF639* (180,524–180,536 kb), *MFN1* (180,549–180,594 kb) and *GNB4* (180,600–180,652 kb) gene region.

Map	# SNPs	χ^2^_1_	−log_10_(pvalue)	Estimated location (kb)	95% confidence interval (kb)	Most significant single SNPs	
						rs	−log_10_(pvalue)
Map 1	49	5.6	1.7	180510.5	180500–180659	rs6794192 c	3.0
Map 2	44	32.00	7.8	180565.8	180505–180655	rs10460887 ^2^	3.3
						rs9822116 ^2^	3.4
						rs17293193 ^2^	3.9
Replication (Hap300)	49	7.6	2.2	180505.3	180500–180653	rs7618348 c	3.95

χ^2^ with 1 degree of freedom from the composite likelihood approach tests for association at estimated location Ŝ_kb_.

rs = SNP identifier ; −log_10_(pvalue) from the most significant SNP within the tested window.

c = single combined p-value for the Map 1, Map 2 and replication samples.

Individual SNPs that showed strongest evidence of association for this window were rs6794192 (180,510,506 bp), rs10460887 (180,538,836 bp), rs9822116 (180,557,316 bp), rs17293193 (180,606,558 bp) and rs7618348 (180,627,432 bp; all *p*-values provided in Table S1). All five SNPs gave low p-values (p<10^−3^) for single-SNP tests of association, with SNPs rs6794192 and rs7618348 genotyped and providing low p-values for all three samples (Map 1, Map 2 and replication) and combined sample single-SNP p-values of 10^−3^ and 10^−4^, respectively (Table 2). An annotated pair-wise LD plot is also presented for this region in Figure S1.

The *MFN1* gene region result replicated for the independent sample of 1430 twins (χ^2^_1_ = 7.6, p = 0.006) using a quantitative test of association, the same analytic LDU window and a different panel of markers for the 3q26 region derived from the Hap300 chip.

### Upstream of *SOX2OT*

**Figure 4:**
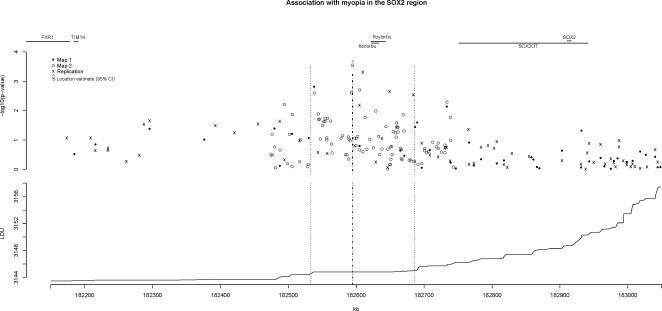
Association with myopia upstream from the *SOX2* region. Legend notes from Figure 3 apply. See Figure S2 for pair-wise LD plot of region.

Analysis of the Map 1 data yielded a significant window that covered the *SOX2OT* gene region (χ^2^_1_ = 7.0, Table 3). Further analysis using the Map 2 data showed a large increase in the significance level (composite likelihood χ^2^_1_ = 46.1, p = 1.1×10^−11^). The physical location for the putative associated common variant in the region using the more informative Map 2 data was estimated to be at 182,595 kb with a 95% confidence interval of 182,533–182,688 kb (Table 3). This location is approximately 156 kb upstream (5′) from the alternate-splicing ncRNA gene *SOX2OT* and 317 kb from the *SOX2* transcription start sites (Figure 4). The confidence interval includes no known genes, but does include two predicted non-coding genes of unknown function (floylorbu 0.51 kb in length and flerlorbu, 21.4 kb [10] and five putative alternative promoters upstream of *SOX2OT*, which between them cover a region of approximately 490 kb [11].

**Table 3:** Localisation of genetic variants upstream from *SOX2* (182,912–182,915 kb), 3q26.

Map	# SNPs	χ^2^_1_	−log_10_(pvalue)	Estimated location (kb)	95% confidence interval (kb)	Most significant single SNP	
						rs	−log_10_(pvalue)
Map 1	44	7	2.1	182533.70	182517–183007	rs1518933	2.8
Map 2	94	46.1	10.9	182594.50	182533–182688	rs733422	2.7
Replication (Hap300)	80	14	3.7	182488.50	182500–182694	rs4855026	3.3

χ^2^ with 1 degree of freedom from the composite likelihood approach tests for association at estimated location Ŝ_kb_.

rs = SNP identifier ; −log_10_(pvalue) from the most significant SNP within the tested window.

Individual SNPs most strongly associated with myopia for this window were rs1518933 (182,538,071 bp), rs733422 (182,604,752 bp) and rs4855026 (182,609,663 bp). Figure S2 provides a pair-wise marker LD plot for the *SOX2* gene region.

These results were also observed for the replication sample using a quantitative test of association with Hap300 SNPs covering the same region (χ^2^_1_ = 14, p = 1.8×10^−4^, Table 3).

The SNP coverage for Map 2 did not include SNPs within or in close proximity of *SOX2* (Figure 4), although the Map 2 evidence for association was based on an LDU analytical window that included the gene. The LDU maps illustrated in Figures 2C and 4 show evidence of multiple recombination hot spots around *SOX2*.

### *PSARL* Region

**Figure 5:**
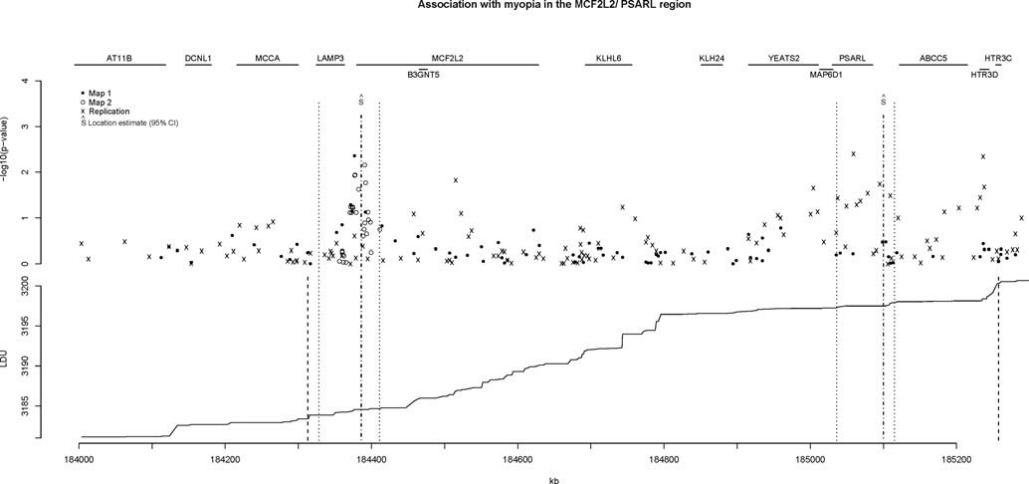
Association with myopia in the *MCF2L2/PSARL* gene region. Legend notes from Figure 3 apply. In this figure, two different location estimates (Ŝ_kb_) and confidence intervals are obtained. One is for the discovery Map 2 data (*MCF2L2* region, to the left of the graph) and the other the replication Hap300 data (*PSARL* region, to the right of the graph). The additional outer vertical dotted lines in the lower LDU graph represent the lower and upper limits of the analytical LDU window used to model the discovery and replication datasets. See Figure S3 for pair-wise LD plot of region.

Preliminary marginal statistical evidence of association for Map 1 data was observed for the analytical LDU window at 184,313–185,257 kb (χ^2^_1_ = 6.0, p = 0.014; Table 4). This region has high recombination rates, is gene rich and includes the genes *LAMP3*, *MCF2L2*, *B3GNT5*, *KLHL6*, *KLH24*, *YEATS2*, *MAP6D1*, *PSARL*, *ABCC5* and *HTR3D* (Figure 5). Based on Map 2 data, the physical location for a putative causal variant was estimated to be at 184,386 kb with 95% confidence intervals 184,356–184,441 kb (χ^2^_1_ = 6.2, p = 0.01; Table 4). This location estimate lies within intron 3 of the 30-exon gene *MCF2L2* with the confidence intervals including *LAMP3* and *MCF2L2*. An annotated pair-wise LD plot is presented for this region in Figure S3.

**Table 4:** Localisation of genetic variants to the *MCF2L2/PSARL* (185,029.882–185,085.356 kb) gene region.

Map	# SNPs	χ^2^_1_	−log_10_(pvalue)	Estimated location (kb)	95% confidence interval (kb)	Most significant single SNP	
						rs	−log_10_(pvalue)
Map 1	70	6	1.8	184371.77	184328–184405	rs512071	2.36
Map 2	28	6.2	2	184386.00	184356–184411	rs534333	2.16
Replication (Hap300)	96	12	3.3	185100.00	185036–185115	rs6775202	2.40

Legend notes from Table 3 apply.

Strong evidence of association to this LDU window was also observed for the replication sample using a quantitative test of association for Illumina Hap300 SNPs genotyped for the same LDU window (χ^2^_1_ = 12, p = 5×10^−4^, Table 4). However, the estimated location for a putative common causal variant for the same window and using Hap300 SNPs was different to that from the discovery SNP coverage (Maps 1 and 2). For Hap300 SNPs, the variant was estimated to be at 185,100 kb (95% CI 185,036–185,115 kb), located in the 3′ UTR of *PSARL*, with the confidence intervals including exons 4–10 of *PSARL* and the 5′ UTR of the neighbouring gene, *ABCC5* (Figure 5). The statistical evidence for the *PSARL* location (χ^2^_1_ = 12) was stronger than that for *MCF2L2* (χ^2^_1_ = 6.2).

### *MYNN*, Downstream (3′) from *SOX2OT* and *LPP* Gene Regions

The estimated locations for common functional variants at these three loci are presented in Table 5. Evidence of association to these loci did not replicate using Hap300 samples, suggesting either Type 1 errors or failure to replicate due to the different genetic coverage of these regions provided by the Map 1 discovery and Hap300 marker sets.

**Table 5:** Localisation of genetic variants to the *MYNN* (170,974–170,988 kb), downstream of *SOX2OT* and *LPP* (189,413–190,080 kb) gene regions, which failed to replicate using Hap300 SNPs.

Map	# SNPs	χ^2^_1_	−log_10_(pvalue)	Estimated location (kb)	95% confidence interval (kb)
***MYNN***					
Map 1	60	8.9	2.6	170941.42	170900–171051
Map 2	30	15.3	4.1	170933.68	170878–170960
Replication	49	2.9	NS	-	-
**Downstream of ** ***SOX2***					
Map 1	33	6.9	2.1	183438.65	183418–183636
Map 2	71	18.6	4.8	183512.17	183439–183634
Replication	40	0.4	NS	-	-
***LPP***					
Map 1	47	8.8	2.5	189342.39	189322–189370
Map 2	46	4.6	1.5	189356.02	189322–189380
Replication (Hap300)	60	0.2	0.1	-	-

Legend notes from Table 3 apply.

## Discussion

Having first replicated the initial linkage to 3q26, the strategy we adopted for fine mapping the large 30 Mb genomic region was to pursue evidence of association in two stages. First, using a high-resolution genetic map, we selected an informative set of SNP markers across the entire region, but at relatively low density to ensure economic feasibility. The second was to follow up those regions that showed strongest evidence of association in the region, with a denser set of markers placed on the same genetic map, on the assumption there are detectable common genetic variants in the region responsible for generating the observed linkage signal.

The approach succeeded, with evidence of replicated association to the *MFN1*, *SOX2OT* and *PSARL* gene regions. It is worth noting that the association initially detected in the three loci regions using Map 1 were only of marginal significance (at p = 0.02, p = 0.008 and p = 0.014, respectively). However, when a higher-resolution map was genotyped for the locus, association was detected at genome-wide significance for *MFN1* and *SOX2OT*.

Evidence of association to the *MCF2L2/PSARL* gene region using Map 2 data remained the same (p = 0.01), but the same LDU window was subsequently replicated more strongly using a different panel of Hap300 SNPs (p = 0.0005). The diverging location estimates in the *MCF2L2/PSARL* region using two different SNP marker sets suggests the possibility of more than one common functional variant and co-incidental association for this LDU window (Figure 5).

The latter emphasises how important informative SNP coverage is for detecting common variants and the use of marker panels that provide similar coverage of local LD patterns. The use of multi-marker tests can efficiently use the LDU locations to provide localization estimates, while for sparse marker sets the use of single SNP tests is likely to result in reduced power to detect association depending upon local LD structure. The results presented here are all the more remarkable in that we were able to replicate the same regions using an unselected sample, for a different panel of SNPs (Hap300) genotyped at different centres.

Some of the regions we have investigated on 3q26 are complicated with high recombination rates or a high density of genes. We have used a model that assumes common susceptibility loci with little or no allelic heterogeneity. As such we recognise there are likely to be more variants and genes in this region that will be identified and replicated by further mapping studies.

The Malecot model delimits the *MFN1* gene region (using the most informative marker set, Map 2) with a 95% confidence interval ranging from 180,505–180,655 kb. Although the strongest evidence of association peaks at 180,565.8 kb in the middle of the *MFN1* gene at exon 7, the confidence interval includes two neighbouring genes, *ZNF639* and *GNB4*.

Mitofusin-1 (Mfn1, the protein derived from *MFN1*) is a mitochondrial outer membrane protein, widely expressed in human tissues but varying in mRNA expression levels between tissues [12]. Mfn appears to be a key player in mediating mitochondrial fusion and morphology in mammalian cells [12]. Its interest as a possible candidate gene involved in ocular function stems from its relationship with *OPA1*, a dynamin-related protein of the inner membrane which is mutated in autosomal dominant optic atrophy [13],[14]. *OPA1* requires Mfn1 to regulate mitochondrial fusion [15]. *OPA1* is expressed in embryonic retina at many levels, not just the ganglion cells leading to the optic nerve, and continues to be expressed in adult retina with unknown function [16].

*GNB4* is of interest as a myopia susceptibility gene, as the Gβ4 protein has been shown to be expressed in retinal ON bipolar cells. The function of bipolar cells in the retina is detection of the edge of objects, and so these cells may be involved in detection of hyperopic blur that is believed to drive the signal for eye growth in myopia. Inhibition of the retinal ON bipolar cells stops the compensatory eye growth when a negative lens or occluder is placed over chick or kitten eyes [17].

*SOX2* is a fundamental homeobox gene, 2 kb in length, involved in ocular development, with mutations leading to anophthalmos [18]. The known interaction between the *SOX2* and *PAX6* genes in lens development suggests the possibility that these may also influence development of refractive error. *PAX6* lies at the centre of our 11p13 linkage signal from the original linkage scan, although we found no intra-genic association with *PAX6* using tagging SNPs [7]. Recent studies illustrate the important role that gene regulatory elements can play in disease susceptibility including for example, a homeobox transcription factor that influences heart development and subsequent risk of atrial fibrillation [19]. There is considerable body of evidence for the role of regulatory elements associated with *PAX6* [20], and on regulatory regions for *SOX2* [21].

*SOX2* itself lies in the intron of another larger (240 kb) non-coding RNA gene *SOX2OT*, which may play a regulatory role in *SOX2* expression [21]. *SOX2OT* is a highly complex locus, which appears to produce several proteins with no sequence overlap, with 14 documented alternative splicing mRNAs, 5 non-over-lapping alternate last exons and 7 validated alternative polyadenylation sites. Upstream of SOX2OT there are also 5 possible alternative promoters [11] (DA281835–DA310380) and two putative ncRNA genes of unknown function, flerlorbu and floylorbu (Figure 4). Whether these elements co-operate with *SOX2OT* in regulating *SOX2* is unknown.

The protein presenilin-associated rhomboid-like protein (PARL, coded for by the gene *PSARL*) is a mitochondrial inner membrane protease, which interacts with *OPA1* to inhibit the mitochrondrial remodelling process that signals apoptosis [22]. This reflects the broader phenomenon that molecular mechanisms behind mitochondrial morphology have been recruited to govern novel functions, such as development, calcium signalling, and apoptosis [23].

PARL plays two important known roles. The enzyme cleaves OPA1 to produce the anti-apoptotic truncated soluble form of *OPA1*, which prevents cristae remodelling and the subsequent release of mitochondrial cytochrome c into the cytosol to stimulate apoptosis. The anti-apoptotic effects of these proteins are independent of mitochondrial fusion [22]. In addition, PARL appears to be implicated in mitochondria-to-nucleus signal transduction - following proteolytic processing of PARL, a small peptide sub-unit (P-beta domain) is released and translocated to the nucleus by an unknown mechanism [24].

The *OPA1* gene encodes a 960 amino acid mitochondrial dynamin-related guanosine triphosphatase (GTPase) protein, which is transported from the nucleus to the outer surface of the inner mitochondrial membrane and interacts with Mfn1 and PARL to cause mitochondrial fusion and suppress mitochondrial-led apoptosis, respectively. OPA1 is widely expressed throughout the body, but most abundantly in the retina, followed by the brain. In the eye, OPA1 is present in the cells of the retinal ganglion cell layer, inner and outer plexiform layers and inner nuclear layer [16].

In summary, we have detected and replicated three novel loci at *MFN1*, *SOX2OT* and *PSARL* using a multi-marker approach that models LD structure. We performed a two-stage design to ensure adequate SNP coverage using a high-resolution LDU map. Prior evidence of replicated linkage to this region means these associations are likely to be real with *MFN1*, *GNB4*, *PSARL* genes and regulatory non-coding RNAs in the vicinity of *SOX2*, all plausible candidates. Although the mechanisms are not clear, this study strongly suggests that two fundamental mitochondrial molecular pathways are implicated in the aetiology of myopia.

We are confident that additional mapping studies for these data are likely to replicate further candidate genes at 3q26-28 and genome-wide, which along with *MFN1* and *PSARL*, can be taken forward to clarify the molecular genetic aetiology of common myopia.

## Materials and Methods

### Subjects

Twins in this study volunteered through media campaigns to be on the TwinsUK Adult Twin Registry at St Thomas' Hospital, London [25]. Subjects were invited to attend the hospital for a visit, which involved collection of multiple phenotypes including measurement of refractive error using non-cycloplegic autorefraction (ARM-10 autorefractor, Takagi Seiko, Japan), as well as venepuncture for blood collection for DNA extraction. For all studies, full informed consent was obtained, and protocols were reviewed by the Local Research Ethics Committee.

A postal enquiry was also initiated in 2002–2003, asking about ocular history and requesting subjects' ocular refraction prescription from their optometrist. We used postal data for those subjects without autorefraction data. Included in the questionnaires were questions about spectacle wear to cross-check refractive error data supplied. Subjects were excluded if they gave a history of cataract surgery, laser refractive surgery, retinal detachment or other ocular problems that might have influenced refractive correction.

Spherical equivalent (SE) was recorded in the standard manner as the sum of the spherical power and half the cylindrical power in diopters (D). The mean SE for left and right eye was calculated for each individual, and where data was available for only one eye, this was used as the SE for the subject.

### Population Stratification

Although there has been little evidence of population stratification in population-based studies of self-reported Britons, we assessed for possible stratification with little or no evidence of stratification observed for these data [26].

### Linkage Replication

We attempted to replicate the evidence from our original genome-wide analysis using AR data [7] for linkage to chromosomes 11p13 (MYP7), 3q26 (MYP8), 4q12 (MYP9) and 8p23 (MYP10) using the same ABI prism microsatellite marker set and Généthon genetic map. Refraction data for an independent sample of 485 DZ twin pairs was obtained from the postal questionnaire refraction data described above. Multipoint genome-wide linkage analyses were performed by use of the unadjusted mean SE of both eyes (in D) and optimal Haseman-Elston regression methods, implemented by use of a generalized linear model [27].

### Association Mapping: Selection of Myopic Cases and Hyperopic Controls for the Discovery Sample

The most informative individuals were selected for genotyping from the lower and upper quartiles of the continuous SE diopter distribution. We selected individuals from a dataset of 915 twin pairs with complete autorefractor data, of whom 431 were monozygotic and 484 DZ pairs, which included the 221 DZ autorefracted pairs from the original linkage study. From the 915 twin pairs, a total of 575 unrelated cases and controls were selected for the discovery sample – 255 monozygotic and 320 DZ singletons. To enrich for genetically informative cases and controls, individuals were selected if they were myopic and had a myopic twin (a “super” case) or alternatively, were hyperopic and had a hyperopic twin (a “super” control). The most myopic individuals (cases), with a diopter score of less than <−1, were selected from each twin pair, where the pair mean was equal to or less than  = <−0.75 diopters. Similarly, the more hyperopic individuals (controls), with a diopter score of at least >+1, were selected from twin pairs with a pair mean greater than >+1 diopters.

This resulted in an ascertained sample, designed to differentially increase allele frequencies between cases and controls for disease susceptibility alleles that predispose individuals to develop myopia. We chose hyperopic rather than normal sighted controls as a strategy to increase power, on the assumption that the aetiology for myopia and hyperopia lie on a continuum between health and disease and that both share genetic risk mechanisms. The discovery data were analysed using case-control status, on the supposition that most of the information would be captured by affection status, but we also tested the case-control data for quantitative association using the original diopter measurements for the selected data.

Case and control samples were simultaneously genotyped using the same platform and arbitrarily allocated to the same plates. Case-control status was independent of plate and well assignment (data not shown).

### LDU Maps and SNP Selection

The LD maps [28] assign markers to locations in linkage disequilibrium units (LDU) that describe the underlying structure of LD in the form of a metric map with additive distances. A high-resolution LDU map for the whole of chromosome 3 was constructed using the CEU PHASE II data from the HapMap Project [29]. The resulting LDU map for 3q26 was used for this study, corresponding to the region with replicated evidence of linkage. The 659 LDU region corresponds to approximately 42.7 cM on the decode linkage map [30], implying that for 3q26, on average ∼15 LDU correspond to 1 cM. For the first part of the project (Map 1), we selected three to four SNPs per 1 LDU across the entire 30 Mb region. This yielded a SNP density of approximately 1 SNP per 15 kb. This selection scheme captured the block-step structure of the high-resolution LD map and ensured good coverage of the LD steps.

Map 1 (the entire 3q26 region) was first partitioned into 51 non-overlapping windows based on the LDU map with a minimum length of 10 LDU per window and by default, not breaking LDU blocks. For the six out of 51 LDU windows showing strongest evidence of association for the Map 1 data (Figure 2), an additional 382 SNPs were genotyped to refine the location estimate (Map 2).

### Replication Data for Association Mapping

Further to the Map 1 and Map 2 studies, we also examined an independent replication sample of 1430 individuals for quantitative association, from which discovery samples and their relatives were excluded. The replication sample was composed of 460 unrelated individual female monozygotic and 338 dizygotic twin singletons and 316 DZ twin pairs. Tests of association were calculated using robust standard errors (clustered by family identifier) to account for relatedness with samples complete for refraction error (autorefractor or postal) and 3q26 genotype data. SNP genotypes for the replication samples were derived from a genome-wide Illumina HumanHap 300 dataset made available at the Twin Research Unit from other studies [26].

To assess the validity of postal SE with AR measures, we compared 138 individuals with both types of measure. The correlation between AR and postal data was 0.93, with a mean difference (AR – postal) of −0.241 (standard deviation = 0.93) and no observed relationship between the differences and the means for the two measures (p = 0.75).

### Genotypes and Quality Control

Discovery Map 1 and Map 2 sample handling, DNA genotyping and genotype calls were performed by Ellipsis Biotherapeutics Corporation (Toronto) using an Illumina Beadstation. SNPs were screened for quality control before analysis and rejected if the marker showed strong evidence of Hardy Weinberg disequilibrium (at a threshold of χ^2^_1_> = 12), SNP-wise missing rates greater than 10% or MAF≤0.01. Samples with a total of more than 30% case-wise missing were also removed before analysis.

For the 3q26 replication sample, all samples were typed using the Infinium assay (Illumina, San Diego, USA) with fully compatible SNP arrays, the Hap300 Duo, Hap300, and Hap550. Quality control measures taken for these data are detailed in [26].

### Association Mapping

Allelic tests of association were initially performed for each marker. The association measure, z, from the 2×2 table between the myopia phenotype (0, 1) and the two alleles of each SNP marker were obtained for Map 1 and Map 2 as z = |D|/f(1−R), where D is the covariance between myopia-status and the marker alleles, *f* is the frequency of myopic individuals in the sample and *R* is the minor allele frequency [31].

The significance of each window (or LDU region) was tested using a composite likelihood approach that simultaneously combines information from all markers within each window [32] on the basis of the Malecot Model. For the *i_th_* SNP, the observed association *z_i_* has an expectation *E*(*z_i_*) estimated by the model as: *E*(*z_i_*) = (*1*−*L*)*Me* ^−*εΔ*(*Si*−*S*)^+*L*. The parameter *M* (intercept) reflects a monophyletic or polyphyletic origin of susceptibility alleles (i.e. proportion of disease alleles transmitted from founders). The parameter *L* (asymptote) is the spurious association at long distance.

The object of LD mapping is to estimate *S*, which is the estimated location of the putative disease gene in the map. The parameter *ε* measures the rate of exponential decline in association with distance and hence *S_i_* is the LDU location of the ith marker. The Kronecker Δ is used for map direction and assures a correct sign, where Δ = 1 if S_i_≥S or Δ = −1 if S_i_<S. Given the observed associations for *z_i_*, the Malecot parameters are estimated iteratively by combining information over all loci within a window. The composite likelihood is calculated as *Λ* = *∑ K_i_* [*z_i_*−*E*(*z_i_*)]*^2^*, where *z* and *E*(*z*) are the observed and expected association values, respectively, at the *i*th marker SNP. Their squared difference is weighted by an information index K_i_, which is estimated as: K_z_ = χ^2^_1_/z^2^, where χ^2^_1_ is the Pearson's χ^2^_1_ from the 2×2 table (myopia status by SNP alleles).

Following Maniatis *et al.* [32], we used two different sub-hypotheses of the model to test for evidence of association. The null hypothesis is model *Null* where *M* = 0. The alternative model *Full* allows the estimation of both *M* and *S*. Hence the contrast between these two models tests for association to a region and for a disease determinant at location *S*. The difference in marker-density between Map 1, Map 2 and the replication samples genotyped for Hap300 SNPs, was taken into account by the use of an F statistic with *df_1_* and *df_2_* degrees of freedom. The degrees of freedom *df_1_* was the number of SNPs minus *df_2_* parameters estimated in the *Full* model. The F-value was estimated as *F*(*df_1_*, *df_2_*) = [(*Λ_Null_*−*Λ_Full_*)/*df_2_*]/*Λ_F_*/*df_1_*. Subsequently, to facilitate model fit comparison between tests with different degrees of freedom, *p*-values from the *F*-statistic were converted to a χ^2^_1_ (full details of methods are presented in [32]). The 95% confidence interval (CI) for the estimated location Ŝ was obtained as: Ŝ±*t SE*, where *t* is the tabulated value of Student's t-test for *df_2_* degrees of freedom and *SE* is the standard error of parameter Ŝ. Estimates of Ŝ in LDU were converted to kb by linear interpolation of the two flanking SNPs.

Same procedures were used for the replication samples (Illumina HumanHap 300, 1430 individuals). However, as the Hap300 chip had been genotyped for a large number of unselected twins, for this analysis we used the quantitative phenotype instead of the case-control status. Therefore the composite likelihood was calculated using the observed regression coefficient (*b_i_*) for each SNP marker (*i*) and the expected *E*(*b_i_*), which was estimated using the Malecot model for every *i_th_* distance in LDU.

### Multiple Testing and Significance Thresholds

For the association mapping study we present nominal p-values that do not correct for multiple testing. We used the following thresholds to indicate statistical significance at each stage:

### Linkage

For the original discovery sample [7] we used a threshold of LOD 3.2 (α≈10^−4^) to indicate genome-wide significance. For evidence of linkage replication presented in this study, we lowered the threshold to LOD 2 (α≈2×10^−3^), since replicating a true initial linkage result for complex traits is recognized to be difficult due to upward bias in discovery sample estimates [33].

### Discovery Association (Case/Control data; LDU Maps 1 and 2)

Based upon Map 1 results, we took forward six LDU windows for further genotyping (Map 2) that corresponded to the six most statistically significant results. For Map 2 we used a threshold of α = 10^−4^, which is conservative, since a Bonferoni correction would provide a threshold of α≈10^−3^ (0.05/51) based upon approximately 51 independent tests (i.e. 51 analytical windows were used to span the 3q26 30 Mb region).

*Replication association* (*SE quantitative trait; Hap300 SNPs*) We attempted to replicate the six analytic LDU windows, with each test window independent of one another. Hence we considered replication using a threshold of α = 10^−2^ (0.05/6) based upon a Bonferoni correction.

### Electronic-Database Information

The URLs for data software presented herein are as follows:

HapMap, http://www.hapmap.org/ (for HapMap data)

## Supporting Information

Figure S1 Pairwise LD plot (D') for the MFN1 gene region (180,400–180,700 kb) using HapMap Phase II SNPs (Build 35, release 21). The top ideogram represents the whole of chromosome 3, with the yellow bar high-lighting the gene region of interest. Below that shows the local physical distance in kb, local coalescent recombination rates (cM/Mb) and gene locations. Note that two annotated hotspots (cM/Mb) either side of the MFN1 gene coincide with the linkage disequilibrium unit (LDU) steps depicted in Figure 3, at approximately 180,500 kb and 180,660 kb.
(0.13 MB DOC)

Figure S2 Pairwise LD plot (D') for the SOX2 gene region (182,500,–183,000 kb) using HapMap Phase II SNPs, showing local physical distance in kb (Build 35), local coalescent recombination rates (cM/Mb) and gene locations. Both the LDU map and the HapMap annotated recombination rates indicate elevated levels of recombination in the SOX2OT gene region, especially downstream. The estimated location for the common functional variant is at approximately 182,600 kb in a ∼155 kb region with extended LD (182,530–182,690 kb).
(0.09 MB DOC)

Figure S3 Pairwise LD plot (D') for the MCF2L2/PSARL gene region using HapMap Phase II SNPs showing local physical distance in kb (Build 35), local coalescent recombination rates (cM/Mb) and gene locations. The estimated physical location for the common functional variant at MCF2L2 based upon linkage disequilibrium unit (LDU) study Map 2 is at approximately 184,390 kb (on the left side of the diagram), while the estimated location at PSARL based on the LDU map for Hap300 marker SNPs is at 185,100 kb (on the right).
(0.09 MB DOC)

Table S1 3q26 HapMap II LDU map.
(2.06 MB XLS)

Table S2 Discovery sample single SNP p-value results (Map 1).
(0.26 MB XLS)

Table S3 Discovery sample single SNP p-value results (Map 2).
(0.05 MB XLS)

Table S4 Replication sample single SNP p-value results (Hap300).
(0.54 MB XLS)
