# Supplementary material for: Identification and Replication of Three Novel Myopia Common Susceptibility Gene Loci on Chromosome 3q26 using Linkage and Linkage Disequilibrium Mapping
Source: PLoS Genet. 2008 Oct 10;4(10):e1000220. doi: 10.1371/journal.pgen.1000220 (PMC2556391; doi:10.1371/journal.pgen.1000220)
Supplement: Figure S1 — Pairwise LD plot (D') for the MFN1 gene region (180,400–180,700 kb) using HapMap Phase II SNPs (Build 35, release 21). The top ideogram represents the whole of chromosome 3, with the yellow bar high-lighting the gene region of interest. Below that shows the local physical distance in kb, local coalescent recombination rates (cM/Mb) and gene locations. Note that two annotated hotspots (cM/Mb) either side of the MFN1 gene coincide with the linkage disequilibrium unit (LDU) steps depicted in Figure 3, at approximately 180,500 kb and 180,660 kb. (0.13 MB DOC) [file pgen.1000220.s001.doc]

# 
