# Supplementary material for: Identification and Replication of Three Novel Myopia Common Susceptibility Gene Loci on Chromosome 3q26 using Linkage and Linkage Disequilibrium Mapping
Source: PLoS Genet. 2008 Oct 10;4(10):e1000220. doi: 10.1371/journal.pgen.1000220 (PMC2556391; doi:10.1371/journal.pgen.1000220)
Supplement: Supplementary file 2 [file pgen.1000220.s002.doc]

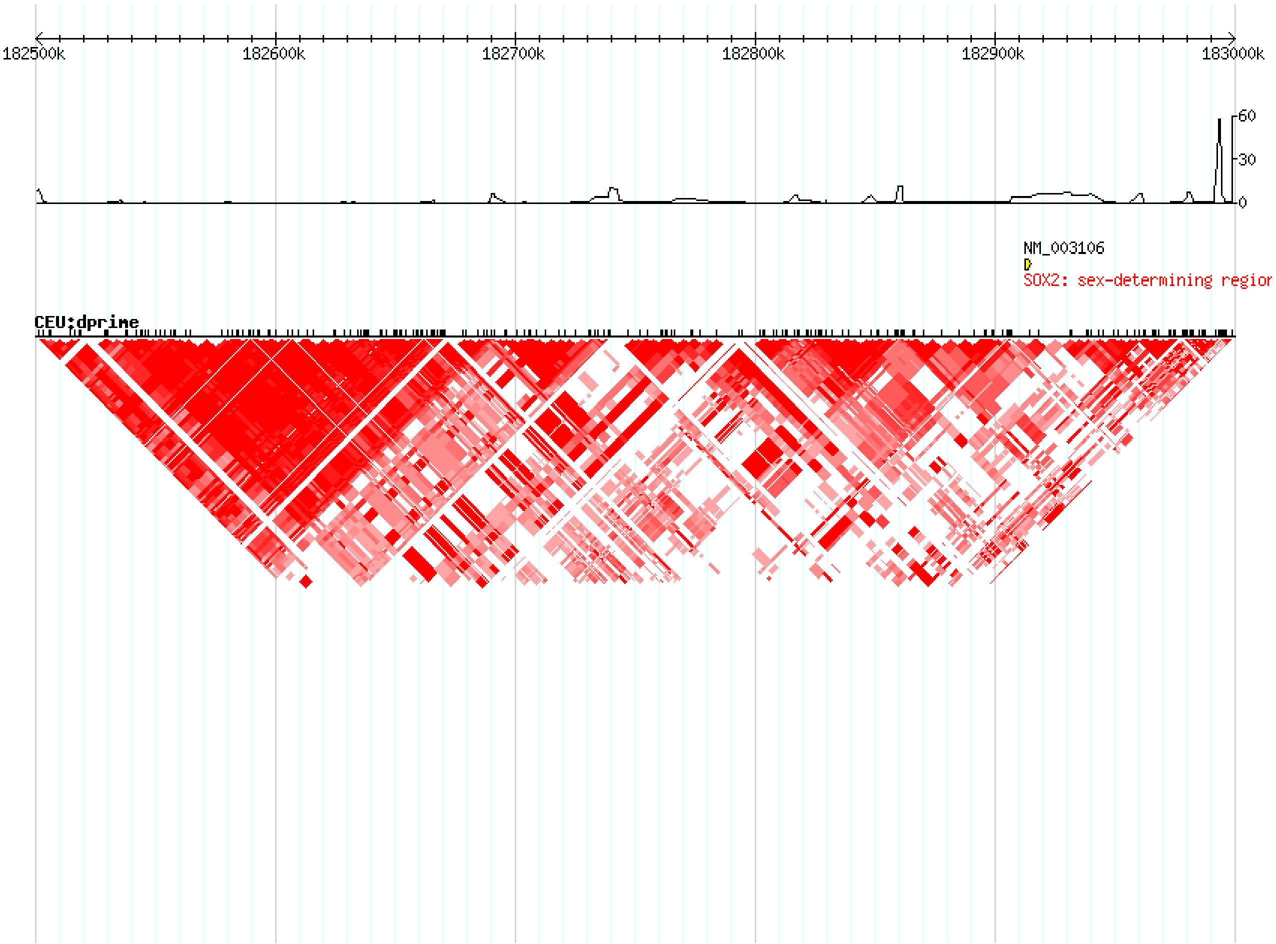

182500k
182600k
182700k
182800k
182900k
183000k
60
30
0
NM_003106
SOX2: sex-determining regior
CEU:dprime
