# Supplementary material for: Identification and Replication of Three Novel Myopia Common Susceptibility Gene Loci on Chromosome 3q26 using Linkage and Linkage Disequilibrium Mapping
Source: PLoS Genet. 2008 Oct 10;4(10):e1000220. doi: 10.1371/journal.pgen.1000220 (PMC2556391; doi:10.1371/journal.pgen.1000220)
Supplement: Supplementary file 3 [file pgen.1000220.s003.doc]

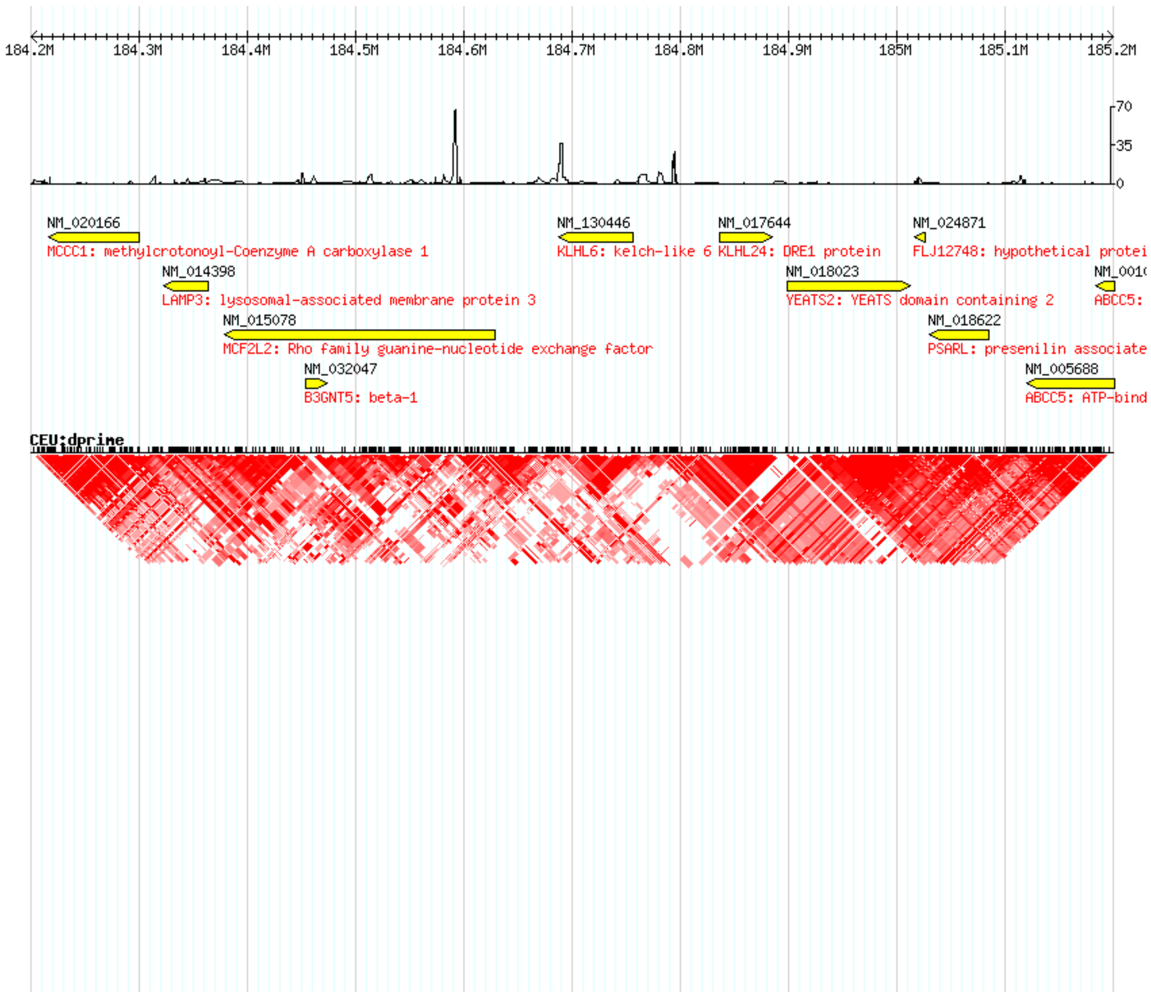

184.2M
184.3M
184.4M
184.5M
184.6M
184.7M
184.8M
184.9M
185M
185.1M
185.2M
70
35
0
NM_020166
MCCC1: methylcrotonoyl-Coenzyme A carboxylase 1
NM_014398
LAMP3: lysosomal-associated membrane protein 3
NM_015078
MCF2L2: Rho family guanine-nucleotide exchange factor
NM_032047
B3GNT5: beta-1
NM_130446
KLHL6: kelch-like 6
NM_017644
KLHL24: DRE1 protein
NM_018023
YEATS2: YEATS domain containing 2
NM_024871
FLJ12748: hypothetical protei
NM_018622
PSARL: presenilin associate
NM_005688
ABCC5: ATP-bind
NM_001(
ABCC5:
CEU:dprime
